# Gleanings from Our Exchanges

**Published:** 1873-12-15

**Authors:** 


The Scientific Congress at Rome.—The Italian savants hold their Congress this year on the 20th inst. in Rome. This is the first time for a great number of centuries, as the organizing committee observes, that reason and science will be able to raise their voices freely in the city of Rome.

The Congress will remain in session ten to fifteen days. Like our scientific congresses it will be divided into sections and classes, comprising all the branches of science. Strangers are received as guests. Senator Tevenzio Mamiani is president; Senator Giuseppe Ponzi and deputy Onovato Cactani assistants. Prof. Luigi Biolchini and Rodolfo Lancaini fill the office of general secretary.—*Gaz. Med. de Paris*, Oct. 18, '73.

The most ingenious explanation ever proffered, perhaps, in explanation of the cause of kleptomania, was that lately stated in New Jersey, "because the individual had been vaccinated with virus from a hooking cow."

### Gleanings from Our Exchanges.

### REPORT ON OTOLOGY.

BY SAMUEL J. JONES, M.D., OF CHICAGO.

[*From Transactions Illinois State Medical Society.*]

In the absence of a report on Otology at the last annual meeting of the Society, your Committee have understood it as the desire of the Society that the present one should embrace the period since the date of the report of the last Committee on Otology appointed by the Society.

The last two years have been prolific of good work in Otology. The amount and character of the contributions to ophthalmology, since the discovery of the ophthalmoscope, have been a gratifying surprise to the profession; and whilst its sister department of otology has scarcely progressed equally with it, yet the recent advance has been most gratifying; and it is probable that in no previous period of two years have the additions been so great as in the last two.

In a previous report, the periodicals devoted to otology were given. They continue to give valuable aid to this department of our profession.

The first American work on "Diseases of the Ear," at all worthy of the subject, appeared last year, of which Dr. Laurence Turnbull is the author. It is a work of great value, not only to the aural surgeon, but to the general practitioner as well. Another work, entitled "On Aural Catarrh and Curable Deafness," by Dr. P. Allen, comes to us from England, and is a valuable contribution in its restricted department of aural practice.

Professor Helmholtz, to whom the profession is indebted for the ophthalmoscope, has recently added to the debt by his investigations in regard to the ear and acoustics, and has recently given us a work on "The Mechanism of the Ossicles of the Ear and Membrana Tympani," which has been translated into our language by Drs. A. H. Buck and N. Smith, of New York. Allied to this department, though not strictly works on otology, are Neftel's small work on "Galvano-Therapeutics," Professor Tyndall's lectures "On Sound," and Cohen's work on "Diseases of the Throat," which are so intimately connected with many affections of the ear, and influence so much the hearing power.

"The American Otological Society" continues to labor for the advancement of this department of surgery, and for the work it has achieved merits commendation. Its contemporary the "Otological Congress" of Germany, thus far has scarcely met the expectation entertained at its organization.

Interesting experiments are being made, and profitable results it is expected will be attained, regarding the influence upon hearing, of the muscles of deglutition, as well as of the tensor tympani and laxator tympani muscles. The physiological use of the different parts of the labyrinth is being investigated, and one result already arrived at is the still further elimination of cases once classed as nervous deafness.

Some singular results have been obtained by division of the semi-circular canals in the lower animals, which seem to throw light on some of those mysterious cases in man wherein cerebral action is disturbed. The microscopical examinations of the labyrinth may further enlighten us as to the function of the different portions, and establish the importance of the so-called member of Corti.

That the ear possesses a certain power of accommodation to sound, seems more than probable, as may be seen by the simple experiment of taking a watch and placing it within the hearing distance of an ear, and gradually removing it farther from the ear until a point shall have been reached which represents its greatest hearing distance. If now the watch be removed still further from the ear, so as no longer to be heard, and then gradually made to approach the ear, it will not be heard until it shall have reached a point nearer the ear than that which registered its greatest hearing distance when receding.

Inquiry is often made by sufferers from impaired hearing as to why they hear better when subjected to loud noises, as in railway travel, when the same noise impairs the hearing of others. This fact seems to derive an explanation from the fact that the accompanying structural change of the ear has so obtunded the organ that the auditory nerve is only excited to action by the stimulus of such pitch and volume of sound as would overwhelm an ear of ordinary sensitiveness: just as the bright rays of the sun would dazzle the sight of a normal eye, whilst the haziness of a diseased cornea would serve as a screen, and limit the amount of light admitted to the eye to what was suited to vision.

Some singular manifestations are observed, at times, in operations upon the ear. Even the introduction of a speculum into the meatus will produce a paroxysm of coughing; probing of the ear to remove secretions therefrom sometimes has the same effect; and the same operation has been known to produce a tingling sensation in the tongue, where there was perforation of the membrana tympani, which tingling disappeared as the inflammation subsided, though the perforation remained. Such a case resulted from scarlatina, and only one ear was affected. On that side there was defective action of the lower lip, and some stammering in speech, which grew less as the disease of the ear improved, and, apparently, as a consequence of such improvement. Such cases show how multifarious are the manifestations of the consequences of acute inflammation of the middle and internal ear; and yet how seldom these cases receive the attention that such grave lesions require! It may not be deemed amiss to introduce here the notes of a few cases that have occurred in the practice of your Committee, in which the results of treatment were as markedly shown as in the above mentioned case:

J. T., aged 17, suffered in infancy from scarlatina, which resulted in perforation of the membrane of the drum of the right ear, and discharge from the ear, which continued almost without interruption for fifteen years. A watch could scarcely be heard when in contact with the affected ear. Thorough cleansing out of the ear by washing out the fauces, Eustachian tube, and middle ear, and filling the meatus with an astringent solution, which was forced through the opening in the membrane of the drum, so as to fill the middle ear and Eustachian tube, at the same time that counter-irritation over the mastoid cells was kept up, resulted in removal of the inflammation, disappearance of the discharge, and marked improvement of the hearing of the ear, which was continued for nearly two years.

H. S., aged 18, also otherwise in good health at the time he applied for treatment, gave as the history of his case, that in infancy he suffered from a "severe cold," which produced great pain, swelling, and discharge, and that the discharge had never ceased from either ear.

Removal of the accumulated and decomposing secretion, which was of very offensive odor, showed on examination that the meatus of each ear was nearly filled with polypoid growths. These were destroyed by caustics, and a solution of sulphate of zinc, with the addition of carbolic acid, was used freely after each cleansing of the ear. Inhalations of a solution of chlorate of potassa was used; and once a day air impressed with iodine was forced, by Politzer's method, through the nose, fauces, Eustachian tube, middle ear, and through the perforated membrane of the drum of each ear, which produced a whistling sound readily recognizable. Within a few days, all odor had disappeared; in a week's time the hearing of each ear improved; and in a month's time conversation in an ordinary tone of voice could easily be heard. In a fortnight more treatment had to be interrupted, in consequence of a change of residence of the patient, but was partially resumed under the care of his family physician; and recent intelligence from the patient reports continued improvement.

Mrs. M., aged 50, applied for treatment, and stated that she did not remember ever to have heard with her left ear; and that recently the hearing of the right one was becoming so impaired as seriously to interfere with her convenience and comfort. Examination showed that the difficulty was chiefly in the Eustachian tubes. Inflation of the tubes by Politzer's method made immediate improvement; and in a fortnight the ticking of a clock could be heard nearly across her parlor, with her right ear closed. Free use of air impressed with iodine, through the Eustachian catheter, gave permanent relief.

H. C. R., aged 38, applied for treatment for an eczematous condition of the meatus of each ear, aggravated at times by formation of small abscesses in the meatus. He stated that for thirty years he had not heard with his left ear. Some disease, of which he knew nothing, had in infancy produced the destruction of his hearing, and he supposed it was permanently lost. Examination of the condition of the ear showed thickening of the membrana tympani and of the middle ear and Eustachian tube; but in time these yielded to treatment, and hearing was restored sufficiently to hear ordinary conversation with that ear, and to recognize the ticking of a watch eight or ten inches distant from it.

R. J. W., aged 35, applied for treatment, and stated that for more than twenty-five years he had not heard with one of his ears, and the hearing of the other was becoming so impaired as seriously to embarrass him in his vocation. In the worse ear, the hearing had been lost so early in life that he did not know the cause. Examination showed the membrana tympani thickened, opaque, and irregularly concave, and the Eustachian tube of that side so closed that no air could be forced through it, either by Politzer's method or by the Eustachian catheter. A few days' treatment made an improvement in each ear; and in one month's time conversation could be heard with the worse ear, and the other was considered entirely restored. The patient also expressed himself relieved of a distressing feeling of fulness and oppression of the head, which had been more embarrassment to him than the impairment of his hearing.

Mrs. F., aged 30, in good general health, applied for relief from distressing tinnitus aurium, which she stated had continued for six years, so that at no time during that period, when awake, was she free from a hissing noise, which she could only compare to the violent escape of steam from an engine. It was so severe as frequently to wake her from sleep at night. Treatment relieved the case, and there has now been almost entire freedom from it, for more than a year.

These are cited to show how much can at times be done to relieve cases which seem so very unpromising as to elicit at least a very guarded prognosis, if not an entirely unfavorable one, and to serve as a further incentive to more thorough revision of the generally accepted views regarding diseases of the ear.

In this connection, your Committee would draw attention to the diagnostic value of thickening of the membrane of the drum. Observation shows that the degree of impairment of hearing is not in proportion to the amount of thickening and opacity of the membrane, contrary to the generally received opinion. Such thickening serves often to aid in forming an estimate of the amount of thickening interior to it, on which the impairment is far more frequently dependent. Nor does it seem of great importance, in treatment, as to what the first cause of such thickening was; but when any thickening has resulted, additions thereto by each succeeding attack of catarrhal inflammation are made. Eruptive diseases are a not unfrequent cause of the beginning of many cases of impaired hearing; but when the acute stage has passed, and some deposit remains, that deposit may be compared to the pebble in the stream, around which accumulations from time to time gather, until what was at first an insignificant matter becomes at a later stage a matter of grave importance, and it is discovered that the most valuable time for successful treatment has passed. In too many cases, that embarrassing consequence, viz.: ringing in the ears, "tinnitus aurium," has resulted. These subjective sounds manifest themselves in various ways, and are often more distressing to the patient than the loss of hearing.

It is important to discover their source before an attempt is made to relieve them. It is necessary to determine whether they originate in a disturbed condition of the equality of the atmospheric pressure on each side of the membrana tympani, and consequent change in the position of that membrane, as often results from closure of the Eustachian tube, or whether there is too great pressure or rigidity within the middle ear disturbing the natural relation of the ossicles with the labyrinth; or whether the lesion is in the internal ear itself —a distinction that is all-important in treatment, for these noises must be regarded as a symptom only, and not the primary difficulty.

Another symptom for it, too — only a symptom ; a consequence, not a disease—is discharge from the ear, "Otorrhea," which requires quite as much care to ascertain its source, as the origin of these sounds for its permanent removal. Of course, the only successful mode of treatment must be removal of the cause which is keeping up the discharge.

Another symptom of affection of the ear, which sometimes is an additional source of distress, is pruritus, or excessive itching of the external meatus. This may be produced by an eczematous condition of the meatus, or in consequence of the presence of the parasite *aspergillus glaucus*, and sometimes, in the milder forms, seems to result from absence of secretion of the cerumen, and accompanying dryness of the lining of the meatus. The treatment must in such case be governed by the cause; but, in nearly all cases, some glycerine and carbolic acid may be added with advantage to the other medicinal substances used—which will generally answer best if used in solution—with which the meatus is to be filled.

The question, "What is earache?" receives such varied answers as to show how indefinite must be the ideas of many as to the character of the lesion of which this is so painful a *symptom*. This pain in the ear may be a neuralgic condition, or may be pain resulting from serious acute inflammation of some part of the ear, in which latter case the treatment required will be wholly different, and on it the life of the sufferer may be staked. Trivial as it may seem from our familiarity with its frequency, its pathology is less understood than it should be. "Earache" suggests at once to the anxious mother of the suffering child "laudanum and sweet oil," and often is even countenanced in this by her family physician, who, without reflection, advises the application of alcohol in the tincture to a surface that is already raw and acutely inflamed. Inspection of the meatus and membrana tympani, in such condition, would convince him of the impropriety of such application; and yet, in what percentage of these cases is such inspection made? Before leaving this subject, your Committee would appeal to the profession in behalf of the little sufferers in these cases, where the pain is so excessive that when it occurs in vigorous adults, they say they can only compare its severity to that produced by bone-felon.

In conclusion, a few words regarding the progress made in the treatment of affections of the ear are offered. As a consequence of recent additions to the minute anatomy of the ear, and its pathology, it would be expected that progress would be made in the results of treatment of its affections. In recording the history of some cases in the earlier part of this report, some allusion was also made to treatment; but some further suggestions are made as to the results attained in that large class of cases so general in this section of our country, viz.: a hardened sclerosed condition of the interior of the middle ear. It may be safely stated that in this part, including with it the Eustachian tube, is where by far the majority of the affections of the ear occur. In many cases the disease originates in this part; in many others it originates in some pharyngitis, and extends through the Eustachian tube, until it involves the entire middle ear, and includes with it the inner layer of the membrana tympani. The stage of the disease—whether acute, sub-acute, or chronic —as well as the degree of structural change of the parts, will modify the treatment and determine the character of the application, as to whether it shall be soothing, sedative, or moderately stimulating, or decidedly stimulating. The affection is generally only local in character, and should be treated chiefly by local applications. In nearly all stages of these affections the application to the pharynx of warmth and moisture—conveniently made by a steam atomizer—and then washing out the Eustachian tube and middle ear, through a Eustachian catheter, with some saline solution, and then making the application of the stimulant, seems to produce the best results. No single remedial agent seems to answer better as a local stimulant in these cases than iodine, which is so volatile that air can readily be impressed with it and forced through a catheter into the middle ear, and even to the mastoid cells, and has the advantage of impressing the whole surface evenly, and not setting into the more dependent portions of the irregular space of the middle ear, there to produce erosion as fluids of an irritating character might do. This course of treatment has shown how incorrect is the impression that cases of thickening of the middle ear—so-called catarrhal—cannot be relieved.

That the galvanic current will yet become a valuable auxilliary in the treatment of these affections, becomes more and more probable each year. Some results given by Neftel, in his article "Electro-Otiatrics" give promise for the future; but as yet our knowledge of galvano-therapeutics must be regarded as very incomplete.

An Improvement on Esmarch's Elastic Bandage.—By W. Harrison Cripps, House-Surgeon to St. Bartholomew's Hospital. —Esmarch's admirable suggestion of using an elastic bandage to exclude the blood before operating on limbs, and the complete success attending it, are now probably well known. The following is a simple modification of his arrangement, by which many yards of elastic bandage may be dispensed with, and it can be easily and quickly applied.

A short india-rubber tube is used, not only to prevent the blood from returning to the limb, but also for the purpose of removing it in the first place. The two ends of an india-rubber tube, twenty-one inches in length, and about three-eights of an inch thick, are bound together with a piece of twine, the whole forming an elastic ring seven inches in diameter. A grooved reel revolving between a double handle completes the necessary apparatus. To apply this to the arm, three or four complete turns of the elastic ring are wound tightly around the hand in such a manner as to include the fingers and thumb, care being taken that the turns lie even and do not cross one another. The reel is then put under the free portion of the ring connecting the upper and lower coil. The reel is passed round and round the limb in an upward direction; thus each coil is unwound from below as another is added above. In this way four tight coils of india-rubber are carried up the limb to any distance required. The degree of tightness can be regulated with the greatest nicety by the distance the reel is drawn from the limb by the bandager.

This method of driving blood from the limb answers perfectly in the arm, and in the lower part of the leg; but in carrying the bandage over the popliteal space, the flexor tendons prevent the artery being effectually compressed. A firm pad in the space would probably answer the purpose.

To remove the bandage, it may either be unrolled by reversing the action of the reel, or the twine connecting its two ends may be cut with scissors.—*London Lancet.*

G. L. Simmons, M.D., in a letter to the *Western Lancet*, speaks as follows regarding this method of suppressing hæmorrhage during operations:

"By the kindness of Mr. T. Smith, who now fills the position of surgeon at St. Bartholomew's Hospital, made vacant by the death of Holmes Coote, I was invited to see him operate in a case where the circulation was suppressed by the elastic bandage. A boy with a diseased tibia was put under the influence of chloroform, the bandage applied, and the diseased bone removed. The experiment was so completely successful that no blood was lost during the operation, all of the tissues being blanched, even to the osseous structure. I need hardly remind you, as remarked by Mr. Smith, 'that this operation is usually made slow and troublesome by the constant welling-up of blood.'

"At King's College Hospital, I saw Mr. Jno. Ward operate in a case of a diseased elbow, where it was necessary to open long and numerous sinuses, and so exsanguined were the tissues that not a drop of blood was evident; and the surgeon stated that he could define the structures as clearly as if a dead limb were under his knife. He also mentioned a case of a late operation upon an arm, very near to the axilla, where he had first forced all of the blood out of the limb by elastic pressure, and had then applied a pad over the axillary artery, keeping it in place by numerous turns of rubber-tubing over the shoulder.

Abuse of Nux-vomica and its Alkaloids. —Dr. Jas. Thompson, of Leamington, writes to the *British Med. Jour.* of Oct. 11, that he is satisfied of an increasing disposition on the part of many people to resort to nux-vomica as a stimulant, and cites a few cases in which the practice had led to serious results.

